# The impact of obstetric interventions and complications on women’s satisfaction with childbirth a population based cohort study including 16,000 women

**DOI:** 10.1186/s12884-019-2633-8

**Published:** 2019-12-11

**Authors:** Maja Falk, Marie Nelson, Marie Blomberg

**Affiliations:** 1 Department of Obstetrics and Gynecology, Linkoping University, 58183 Linkoping, Sweden; 2 Department of Clinical and Experimental Medicine, Linkoping University, 58183 Linkoping, Sweden

**Keywords:** Birth satisfaction, VAS, Visual analog scale, Mode of childbirth, Cesarean, Induction

## Abstract

**Background:**

As a quality marker and a tool for benchmarking between units, a visual analogue scale (VAS) (ranging from 1 to 10) to estimate woman’s satisfaction with childbirth was introduced in 2014. This study aimed to assess how obstetric interventions and complications affected women’s satisfaction with childbirth.

**Methods:**

A retrospective cohort study including 16,775 women with an available VAS score who gave birth between January 2016 and December 2017. VAS score, maternal and obstetric characteristics were obtained from electronic medical records and crude and adjusted odds ratios (aOR) were calculated.

**Results:**

The total prevalence of dissatisfaction with childbirth (VAS 1–3) was 5.7%. The main risk factors for dissatisfaction with childbirth were emergency cesarean section, aOR 3.98 95% confidence interval (CI) 3.27–4.86, postpartum hemorrhage ≥2000 ml, aOR 1.85 95%CI 1.24–2.76 and Apgar score < 7 at five minutes, aOR 2.95 95%CI 1.95–4.47. The amount of postpartum hemorrhage showed a dose-response relation to dissatisfaction with childbirth. Moreover, labor induction, instrumental vaginal delivery, and obstetric anal sphincter injury were significantly associated with women’s dissatisfaction with childbirth. A total number of 4429/21204 (21%) women giving birth during the study period had missing values on VAS. A comparison of characteristics between women with and without a recorded VAS score was performed. There were statistically significant differences in maternal age and maternal BMI between the study population and excluded women due to missing values on VAS. Moreover, 64% of the women excluded were multiparas, compared to 59% in the study population.

**Conclusions:**

Obstetric interventions and complications, including emergency cesareans section and postpartum hemorrhage, were significantly related to dissatisfaction with childbirth.

Such events are common and awareness of these associations might lead to a more individualized care of women during and after childbirth.

## Background

The woman’s experience of childbirth is an important quality measure in obstetric care. A negative or traumatic birth experience may have both immediate and long-term effects on the mother and the child. It has shown to be a risk factor for developing postpartum depression [1] and maternal fear of childbirth [2]. A previous traumatic childbirth experience and fear of childbirth were also strong predictors of a mother’s request for elective cesarean section (CS) in subsequent births [3, 4].

Different measurements have been used for assessing women’s subjective experiences of childbirth. Questionnaires is the most commonly used method so far and several validated questionnaires have been developed through the years. Wijma Delivery Expectancy/Experience Questionnaire (W-DEQ) has been used in a number of studies for evaluating fear and experience of childbirth [5]. Small or non-representative samples, inclusion of only healthy women, poor reliability and validity and lack of intern consistency have been discussed as possible bias with the questionnaires [6]. Qualitative studies with semi-structured interviews have also been used to explore women’s experiences of delivery. For instance, Hauck and colleagues interviewed 20 women to investigate if childbirth expectations influenced women’s experiences of labor. The authors concluded that expectations were highly related to satisfaction, but that supportive caregivers and involvement in decision-making could improve the experience regardless of whether the expectations were fulfilled or not [7].

The Visual Analogue Scale (VAS) is a psychometric measurement that has been used frequently in pain measuring. In addition, VAS has been used to evaluate patient satisfaction in care, for instance in different kinds of surgery [8]. Larsson et al. used VAS along with several questionnaires to explore factors associated with a negative childbirth experience. A secondary aim of that study was to compare VAS with the questionnaire W-DEQ. The results showed a moderate but significant correlation between the two measurements [9].

Many factors influence the woman’s birth experience. Previous studies have found an association between a negative childbirth experience and a lack of support from the midwife [10, 11]. Similarly, Hodnett found that the amount of support from the caregivers and the caregiver-patient relationship were two of the most important factors contributing to women’s experiences of delivery [12]. Other factors often mentioned as risk factors for a negative birth experience are fear of childbirth [13], pain [9] and lack of control [10]. In a thematic analysis, information was collected from women with a negative birth experience; ‘Complications for mother, child or both’ emerged as one of the main themes [11]. Ulfsdottir et al. found that a low Apgar score at 7 min was a risk factor for a negative birth experience [14]. A recent systematic review demonstrated that the impact of postpartum hemorrhage (PPH) on the woman’s physical and emotional well-being was largely unknown [15]. The association between experience of childbirth and use of epidural anesthesia is debated. Ulfsdottir et al. identified the use of epidural anesthesia to be a risk factor for a negative childbirth experience [14]. Other studies found no association between maternal satisfaction and epidural anesthesia [12, 16]. The incidence of obstetric anal sphincter injury (OASI) has increased over the past decades in the Nordic countries [17]. OASI has been associated with perineal pain [18] and anal incontinence [19]. Although complications have been identified and investigated, the impact of OASI on short term maternal satisfaction is less explored. In a Swedish qualitative study of 1248 women diagnosed with an OASI, the participants described their complications the first two months after birth as “a worse nightmare than expected” [20]. Pain, incontinence, mental distress and dyspareunia were frequently mentioned by the women. The influence of mode of birth on the woman’s experience of childbirth is disputed. Several authors have suggested that women who have an unplanned delivery are less satisfied than women who have a planned delivery [21, 22]. On the other hand, Hodnett stated that medical interventions did not affect the woman’s experience of childbirth as much as the attitude and behavior of the caregiver [12]. Moreover, a number of studies suggested there was no association between mode of delivery and satisfaction of childbirth [9, 14]. Nevertheless, the emergency CS is well agreed to be significantly associated with a more negative experience of birth compared to other modes of birth [21, 23]. The birth experience regarding elective CS is also debated. Previous investigations have indicated a better experience among women who had an elective CS compared to vaginal delivery [7]. Bryanton et al. did not support this finding [24]. The authors claimed that women who had an elective CS were less satisfied than those who had an emergency CS or vaginal birth. Furthermore, the study concluded that being with their infant the moment after delivery had a greater impact on the perceived experience, regardless the mode of birth.

Obviously, the number of obstetric interventions such as induction of labor and CS are constantly increasing worldwide [25]. Therefore this study aimed to assess in a large data set whether obstetric interventions, mode of birth and obstetric complications affected women’s satisfaction with childbirth.

## Methods

**Fig. 1:**
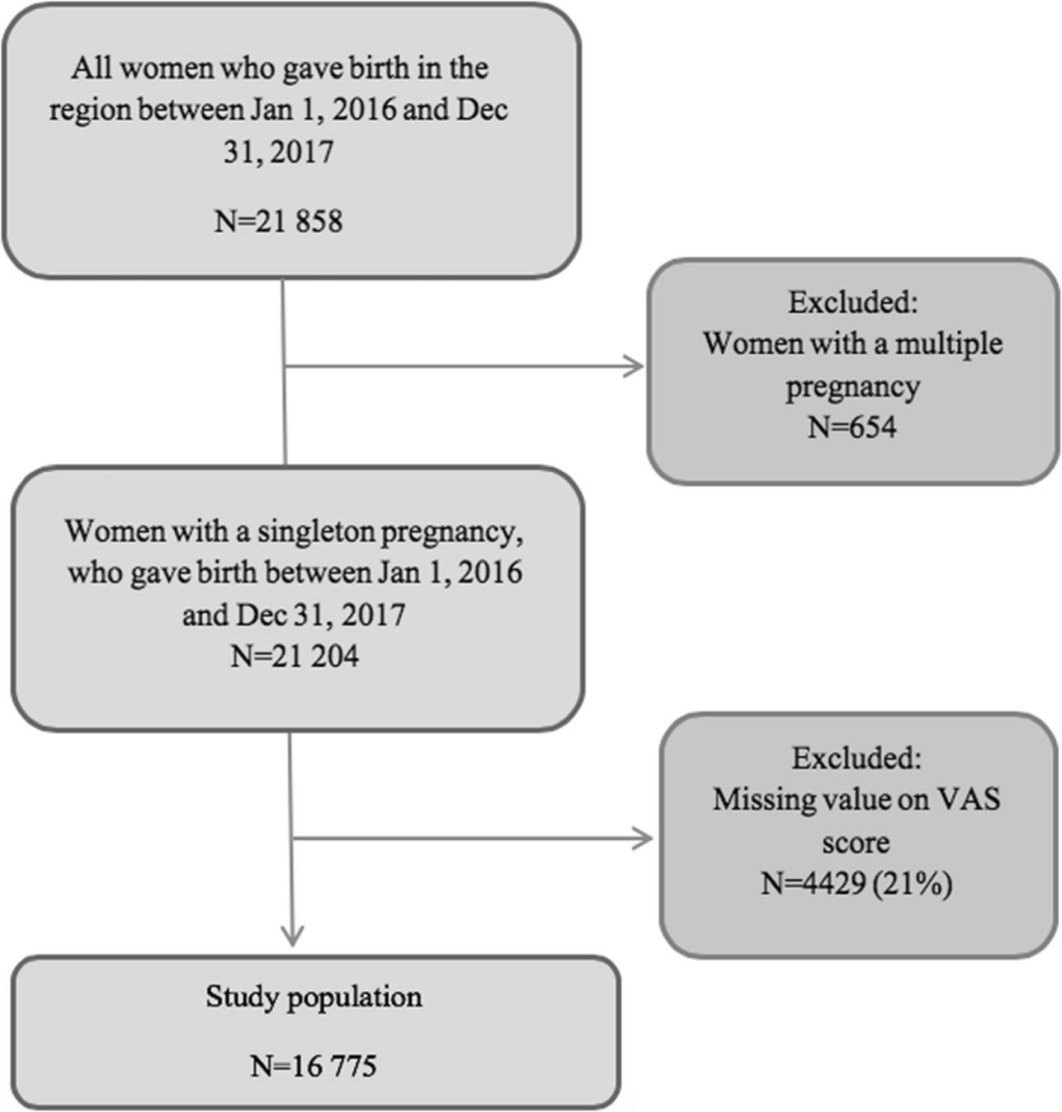
Flow chart of the study population

This retrospective observational cohort study was conducted between January 1, 2016 and December 31, 2017 at seven delivery units in the southeast region of Sweden and included all women with singleton births during the study period. At the start of the study period (2016) the VAS estimation of childbirth satisfaction was well established in routine clinical care and had been practiced in nearly two years. A flow chart of the study population is presented in Fig. 1.

Assessment of satisfaction with childbirth is routinely estimated using a visual analog scale, ranging from 1 to 10, where 10 is most satisfied. There is a written clinical guideline on VAS estimation. The women are asked by the midwife working at the postnatal ward (not involved in the labor or birth) to assess their overall satisfaction with childbirth. The assessment is made after going through the course of the childbirth. The woman is able to ask questions, and get information about why obstetric interventions were made or not. The VAS assessment is made at the end of the dialog. This dialog are performed before discharge and therefore the average time between childbirth and VAS assessment is usually1–2 days. The reported VAS value is then documented in the woman’s electronic medical records (EMR). Usually a VAS scale ranges from 0 to 10 but due to an electronic medical record system error missing values on VAS are marked as 0. Therefore, 0 could not be used as a patient reported VAS score. VAS scores were extracted from women’s EMRs as the main outcome variable in the present study. The VAS score was further dichotomized into dissatisfaction (VAS 1–3) and not being dissatisfied with childbirth (VAS 4–10). This categorization was based on the current clinical recommendation to offer extra psychosomatic support to women scoring VAS 1–3, in order to enable their processing of a putative traumatic experience.

Demographic data were collected from the EMR. Maternal characteristics extracted were maternal age, body mass index (measured upon enrollment at the antenatal care center) and parity. Maternal age was coded as < 25 years; 25–35 years; > 35 years. Body Mass Index (BMI) was coded according to the WHO classification of adult weight: underweight (< 18.5 kg/m^2^), normal weight (18.5–24.9 kg/m^2^), overweight (25–29.9 kg/m^2^), obesity class I (30–34.9 kg/m^2^), obesity class II (35–39.9 kg/m^2^), and obesity class III (> 40 kg/m^2^) [26]. Parity was recoded into primiparas and multiparas.

Obstetric exposure variables extracted from the EMR were onset of labor, epidural anesthesia, labor augmentation (oxytocin), mode of birth, OASI, PPH and Apgar score at five minutes. PPH was coded as < 500 ml; 500–999 ml; 1000–1999 ml; ≥2000 ml, and Apgar score at five minutes was coded as < 4; < 7; ≥7. Epidural anesthesia and OASI were dichotomized into (yes or no). Onset of labor was divided into spontaneous, induction and elective CS. Mode of birth was categorized into four groups: normal vaginal birth, instrumental vaginal delivery (vacuum extraction or forceps), elective CS and emergency CS.

Obstetric interventions and complications were then related to dissatisfaction with childbirth (VAS 1–3).

### Statistical analysis

Data were analyzed using IBM SPSS Statistics for Windows, Version 24.0. Armonk, NY: IBM Corp. Descriptive statistics were presented as mean score, standard deviation, and absolute and relative frequency. Maternal characteristics were evaluated using chi-squared tests for categorical variables and t-tests for numerical normally distributed variables. Women with and without a documented VAS score were compared concerning certain characteristics. Reference categories for the analyses of obstetric variables were chosen as follows: spontaneous onset of labor, no epidural anesthesia, no labor augmentation (oxytocin), normal vaginal birth, no OASI, PPH < 500 ml and Apgar ≥7. Crude and adjusted odds ratios (ORs and aORs) were calculated with 95% confidence intervals (CI) using univariable and multivariable logistic regression. In the multivariable analyses, adjustments were only made for maternal characteristics statistically significantly associated with birth satisfaction. Epidural anesthesia was further adjusted for mode of birth. All analyses were two-sided, and *P*-values less than 0.05 were considered as statistically significant.

This study was approved by the Regional Ethical Review Board in Linköping,Sweden (Dnr 2018/337–31).

## Results

A total number of 21,204 women with a singleton pregnancy gave birth between Jan 12,016 to December 31, 2017. A missing value in the EMR on VAS occurred in 4429 women leaving 16,775 as the final study population. In the study population, 69% (11,493/16775) of the women were very satisfied with childbirth and chose the three highest VAS scores (8, 9 or 10). However, 953 (5.7%) women reported VAS 1–3. Among the 16,775 women included in this study, the mean VAS score was 7.94 (SD 2.1). The mean maternal age was 29.7 years (range 14–49 years, SD 5.0). Forty percent (*n* = 6632) of the women were primiparas and 54% (*n* = 8722) were classified as normal weight (BMI 18–24.9 kg/m^2^). Table 1 presents a cross-tabulation of maternal characteristics in the study population according to satisfaction with childbirth.

**Table 1:** Maternal characteristics of the study population. In comparison between women with dissatisfaction with childbirth (VAS 1–3) and women not being dissatisfied (VAS 4–10). Chi-squared tests were used for categorical variables and t-tests for numerical variables

	Dissatisfaction with childbirth VAS 1–3 (*n* = 953)	Not being dissatisfied with childbirth VAS 4–10 (*n* = 15,822)	P-value
Maternal age (years)			
Mean [SD]	30.2 [5.0]	29.6 [5.0]	0.001*
< 25	101 (10.6)	2237 (14.1)	0.006*
25–35	700 (73.5)	11,327 (71.6)	
> 35	142 (14.9)	2098 (13.3)	
Missing n	10	160	
Body Mass Index			
< 18.5	14 (1.5)	365 (2.4)	0.079
18.5–24.9	469 (49.2)	8253 (54.3)	
25–29.9	274 (28.8)	4212 (27.7)	
30–34.9	107 (11.2)	1669 (11.0)	
35–39.9	42 (4.4)	542 (3.6)	
> 40	15 (1.6)	185 (1.2)	
Missing n	32	596	
Parity			
Primiparas	499 (52.4)	6133 (38.8)	< 0.001*
Multiparas	438 (46.0)	9468 (60.0)	
Missing n	16	221	

VAS: visual analog scale. Categorical data are presented as number and (%). *P-values < 0.05 were considered as statistically significant

No association was found between BMI and satisfaction with childbirth. Likewise, no difference in satisfaction with childbirth was shown when comparing normal weight women with the rest of the women in the study sample (*p* = 0.052). Primiparas and women > 35 years were more likely to report dissatisfaction.

The risk of dissatisfaction with childbirth in relation to obstetric interventions before birth are shown in Table 2

**Table 2:** Obstetric interventions and risk of dissatisfaction with childbirth. Logistic regression analyses were used to estimate crude and adjusted odds ratios (ORs and aORs) with 95% confidence intervals (CI)

	Total number of births *N* = 16,775	Dissatisfaction with childbirth VAS 1–3		
		Number n (%)	Crude OR (95% CI)	Adjusted OR (95% CI) ^a^
Onset of labor				
Spontaneous	13,071	669 (5.1)	ref.	ref.
Induction	2655	232 (8.7)	1.78 (1.52–2.07)	1.69 (1.44–1.98)
Elective CS	1049	52 (5.0)	0.97 (0.72–1.29)	1.00 (0.74–1.34)
Epidural anesthesia				
Yes	6074	509 (8.4)	2.11 (1.85–2.41)	1.90 ^b^ (1.64–2.20)
No	10,701	444 (4.1)	ref.	ref.
Oxytocin augmentation				
Yes	6455	561 (8.7)	2.41 (2.11–2.75)	2.11 (1.83–2.44)
No	10,320	392 (3.8)	ref.	ref.

OR: odds ratio; CI: confidence interval; CS: cesarean section. ^a^Adjusted for maternal age and parity. ^b^ Adjusted for maternal age, parity and mode of birth

Induction of labor (aOR 1.69, 95% CI 1.44–1.98), epidural anesthesia (aOR 1.90, 95% CI 1.64–2.20) and oxytocin augmentation (aOR 2.11, 95% CI 1.83–2.44) were found to be risk factors for dissatisfaction with childbirth after adjusting for age and parity. When epidural anesthesia was further adjusted for mode of birth, the intervention was still a significant risk factor for dissatisfaction with childbirth (aOR 1.75, 95% CI 1.50–2.04).

The results from the analyses of mode of birth are presented in Table 3.

**Table 3:** Mode of birth and risk of dissatisfaction with childbirth. Logistic regression analyses were used to estimate crude and adjusted odds ratios (ORs and aORs) with 95% confidence intervals (CI)

	Total number of births N = 16,775	Dissatisfaction with childbirth VAS 1–3		
		Number n (%)	Crude OR (95% CI)	Adjusted OR (95% CI) ^a^
Normal vaginal birth	13,990	625 (4.5)	ref.	ref.
Instrumental vaginal delivery	852	119 (14.0)	3.47 (2.81–4.28)	2.89 (2.32–3.60)
Elective CS	1049	52 (5.0)	1.11 (0.83–1.49)	1.12 (0.83–1.50)
Emergency CS	884	157 (17.8)	4.62 (3.82–5.59)	3.98 (3.27–4.86)

OR: odds ratio; CI: confidence interval; CS: cesarean section. ^a^Adjusted for maternal age and parity

Emergency CS was the strongest predictor of reporting dissatisfaction with childbirth (aOR 3.98, 95% CI 3.27–4.86). Similarly, an instrumental vaginal delivery was a risk factor for dissatisfaction with childbirth (aOR 2.89, 95% CI 2.32–3.60), compared to a normal vaginal birth. No significant association was found between elective CS and dissatisfaction with childbirth (aOR 1.12, 95% CI 0.83–1.50), using normal vaginal birth as a reference. Obstetric complications after birth in relation to dissatisfaction with childbirth are presented in Table 4.

**Table 4:** Obstetric complications and risk of dissatisfaction with childbirth. Logistic regression analyses were used to estimate crude and adjusted odds ratios (ORs and aORs) with 95% confidence intervals (CI)

	Total number of births N = 16,775	Dissatisfaction with childbirth VAS 1–3		
		Number n (%)	Crude OR (95% CI)	Adjusted OR (95% CI) ^a^
Obstetric anal sphincter injury				
Yes	390	53 (13.6)	2.71 (2.01–3.64)	2.07 (1.51–2.83)
No	16,384	900 (5.5)	ref.	ref.
Missing n	1			
Postpartum hemorrhage				
< 500 ml	11,876	543 (4.6)	ref.	ref.
500–999 ml	3725	288 (7.7)	1.75 (1.51–2.03)	1.65 (1.42–1.92)
1000–1999 ml	815	82 (10.1)	2.34 (1.83–2.98)	2.11 (1.65–2.72)
≥ 2000 ml	161	27 (16.8)	4.21 (2.76–6.42)	4.11 (2.68–6.30)
Missing n	198			
Apgar score at 5 min				
< 4	19	3 (15.8)	3.00 (0.88–10.28)	2.57 (0.74–8.89)
< 7	179	28 (15.6)	3.16 (2.10–4.76)	2.95 (1.95–4.47)
≥ 7	16,559	918 (5.5)	ref.	ref.
Missing n	37			

OR: odds ratio; CI: confidence interval. ^a^Adjusted for maternal age and parity

If an OASI was diagnosed the risk for dissatisfaction with childbirth was doubled. PPH of all degrees was also significantly associated with a negative experience, compared with bleeding < 500 ml. The adjusted analyses indicated a dose–response relation between amount of bleeding and dissatisfaction with childbirth, the more severe the bleeding, the greater the risk of dissatisfaction. Likewise, the immediate well-being of the infant seemed to highly influence the woman’s reported VAS score. Apgar score < 7 at five minutes after birth was found to be statistically significantly associated with dissatisfaction with childbirth (aOR 2.95, 95% CI 1.95–4.47), compared with the reference category Apgar ≥7. Apgar score < 4 was not related to dissatisfaction with childbirth but low numbers were included in that analysis.

Due to 21% (4429/21204) missing values on VAS in women giving birth during the study period a comparison of available characteristics between women with and without a recorded VAS score was performed. The results of the analyses are presented in Table 5.

**Table 5:** Comparison between women with and without a documented VAS score. Chi-squared tests were used for categorical variables and t-tests for numerical variables

	Study population (N = 16,775)	Women excluded (*n* = 4429)	P-value
Maternal age (years)			
Mean [SD]	29.7 [5.0]	29.6 [5.4]	0.377
< 25	2338 (14)	740 (17)	< 0.001*
25–35	12,027 (72)	2900 (66)	
> 35	2240 (13)	638 (14)	
Missing	170 (1)	151 (3)	
Body Mass Index (kg/m^2^)			
Mean [SD]	25.3 [4.9]	25.8 [5.1]	< 0.001*
< 18.5	379 (2)	126 (3)	< 0.001*
18.5–24.9	8722 (52)	2041 (46)	
25–29.9	4486 (27)	1220 (28)	
30–34.9	1776 (11)	521 (12)	
35–39.9	584 (3)	184 (4)	
> 40	200 (1)	69 (2)	
Missing	628 (4)	268 (6)	
Parity			
Primiparas	6632 (40)	1491 (34)	< 0.001*
Multiparas	9906 (59)	2825 (64)	
Missing	237 (1)	113 (3)	

Categorical data are presented as number and (%).*P-values < 0.05 were considered as statistically significant

The mean age of the study population (29.7 years) was similar to the mean age of the women without VAS (29.6 years) (*p* = 0.377). The mean BMIs were also comparable between the groups (25.3 versus 25.8 kg/m^2^), although the difference was statistically significant (*p* < 0.001). Moreover, 64% of the women excluded were multiparas, compared to 59% in the study population (p < 0.001).

## Discussion

This large cohort study showed that obstetric interventions and complications were strongly related to women’s satisfaction with childbirth. The strongest risk factors for dissatisfaction were emergency CS, instrumental vaginal delivery, PPH and Apgar score < 7 at five minutes. Furthermore, induction of labor, epidural anesthesia, oxytocin augmentation and OASI were significantly associated with women’s dissatisfaction with childbirth.

Mode of birth did highly influence women’s satisfaction with childbirth. Emergency CS and instrumental vaginal delivery were strongly related to a reported dissatisfaction with childbirth. These findings are in concordance with the existing literature [10, 21–23]. Although, one study pointed out the importance of separating forceps and vacuum extraction when evaluating birth experience based on findings of an increased risk of post-traumatic symptoms in women who had a forceps-assisted vaginal birth, but no such increased risk after vacuum extraction [27]. Since forceps deliveries are practically non existing in the Southeast region of Sweden our observed relation between instrumental delivery and a negative birth experience could be equalized to vacuum extraction. Prior research has suggested lack of control, insufficient involvement in decision-making and complications for mother or child as potential risk factors for a negative experience of childbirth [16, 27]. These factors may explain the lower degree of satisfaction following an unexpected intervention such as instrumental vaginal delivery or emergency CS. Interestingly elective CS did not protect against dissatisfaction with childbirth when comparison was made with normal vaginal birth. This result contradicts prior research, which has described elective CS as being related to a better birth experience [21]. In the present study, Apgar score < 7 at five minutes and PPH ≥ 2000 ml were highly associated with dissatisfaction with childbirth. These findings further strengthen the suggestion that “complications for mother or child” have a great impact on maternal satisfaction [11]. There was a dose-response relation between degree of PPH and dissatisfaction with childbirth. Lower satisfaction among women with PPH regardless of degree implies that this complication may be even more traumatic for the women than the caregivers are aware of. The explanation for the lower satisfaction could be the loss of consciousness and the reaction to the blood, from both the woman and her partner. Other reasons may also include additional interventions, such as pain, suturing, blood transfusions, anemia and prolonged recovery after birth. The risk of dissatisfaction with childbirth was doubled when suffering an OASI. A follow-up study showed poorer quality of life 10 years postpartum among women suffering an OASI, compared to women without an OASI [28]. To our knowledge, no previous study has focused on the relationship between OASI and women’s overall satisfaction shortly after birth. An OASI may be traumatic for the mother at an early stage, since it often leads to additional immediate interventions such as pain analgesia and suturing. Worries about future problems due to the injury may also reduce satisfaction. There is an ongoing discussion whether extra follow-up of these women should be offered.

The finding of epidural anesthesia to be a risk factor for dissatisfaction with childbirth corresponds with results from Ulfsdottir et al. and Waldenström et al. [10, 14]. In contrary, Hodnett and Carquillat et al. found no significant association between epidural and birth experience [12, 16]. The relationship between birth satisfaction and pain analgesia is complex. Women are more likely to receive analgesia when time in labor is longer or more complicated and this should be kept in mind when the results of this study are interpreted [29]. Another possible explanation for lower satisfaction in women receiving an epidural might be selection bias. Stadlmayr and colleagues stated that a request for epidural anesthesia was related to high levels of physical discomfort and low emotional adaptation [30]. Induction of labor is a common obstetric intervention, with an increasing rate over the last few decades in Sweden. Our results showed that women who were induced were at higher risk of experiencing the childbirth in a negative way. In agreement with our findings, studies have reported a significant association between labor induction and a negative experience of childbirth [10, 31]. However, a recent systematic review of qualitative studies conclude that the experiences of women with induced labor can likely be improved by supporting their informed choice and shared decision making, by giving high-quality, unbiased information about IOL, alternative options, and potential outcomes [32].

The present study has certain strengths. To our knowledge, this study has the largest number of women by the time of date compared to previous studies in the same field, which gave the study sufficient power to evaluate satisfaction with childbirth according to labor characteristics. The broad inclusion of not only healthy women in seven different hospitals reduced the risk of selection bias. Furthermore, the high compliance of VAS (79%) increases the likelihood of the study sample being representative. These advantages make the results of this study likely to be generalizable to other populations in high-income countries. Although there were statistically significant differences between women with and without a recorded VAS score, no clinically relevant differences appeared. Another advantage of this study is that all variables studied in relation to birth satisfaction were prospectively documented in the EMR and therefore recall bias could be avoided. Furthermore, the large sample size made it possible to study rare outcomes in relation to satisfaction with birth, such as low Apgar score and OASI. The process of estimating satisfaction with childbirth using VAS was clinically well established at the time of the study start. A midwife who was not a part of the care team during labor and childbirth evaluated woman’s satisfaction. Consequently, it was possible for the woman to give honest responses to the VAS assessment, without fear of hurting anyone’s feelings. VAS has been validated concerning birth experience by comparing VAS with the W-DEQ questionnaire (The Wijma Delivery Expectancy/Experience Questionnaire) [5] for assessing experience of childbirth. The results showed a significant correlation between the two measurements and that VAS was easier to use than the questionnaire [9].

The present study has several limitations. The purpose of this study was to evaluate how obstetric interventions and complications affected women’s satisfaction with childbirth overall. Focusing on clinically relevant groups, for example women with PPH or women with induced labor, not necessarily to imply causality between the exposure and the outcome. With a purpose (not ours) to come as close as possible proving causality between exposure and outcome controlling for all putative variables would have been correct. This is two different approaches in epidemiology and as far as we know both are acceptable and widely used. We think our data might be useful when informing a women asking for induction of labor that compared with women with spontaneous onset, the risk for dissatisfaction is increased, not saying that the relationship is causal (having taken into consideration all complications that can occur).

Another limitation is that only factors available in the EMR could be evaluated. There might be other putative confounding factors (not available in the EMR) such as socioeconomic status that could have affected our results. Our definition of dissatisfaction (VAS 1–3) was based on the current clinical guideline to offer extra psychosomatic support to women scoring below 4. Another definition might have been correct and rendered other results. The point in time when the satisfaction with childbirth was measured can also be debated. In this study, the measuring of satisfaction occurred just a few days after childbirth. Soet el al stated that initial positive feelings towards giving birth might influence the women’s rating of satisfaction [33]. This was possible for the women included in our study. The high compliance and the equal evaluation of all women included in the study may overcome the possible shortcoming of immediate assessment. Another major limitation is that we have no knowledge about factors that could have positively influenced women’s estimated satisfaction with birth such as caregiver support and involvement in decision-making [7].

## Conclusions

Obstetric interventions and complications, including emergency CS, instrumental vaginal delivery, PPH and Apgar < 7 at five minutes were significantly related to dissatisfaction with childbirth. Other variables significantly associated with dissatisfaction included labor induction, epidural anesthesia, oxytocin augmentation, and OASI. There might be clinical implication of these findings. Women requesting for labor induction or elective CS could be informed that the overall satisfaction with childbirth was not better or even less after these interventions compared to spontaneous onset of labor and normal birth. Secondly, our findings might increase staff’s awareness of the negative impact of relatively common obstetric interventions and complications; one is PPH, on women’s satisfaction with childbirth.

## Data Availability

The datasets used and/or analyzed during the current study are available from the corresponding author on reasonable request.

## Abbreviations

BMI: Body Mass Index
CS: Cesarean section
EMR: Electronic medical records
OASI: Obstetric anal sphincter injury
PPH: Postpartum hemorrhage
VAS: Visual analogue scale
